# Long-Term Aspartame Administration Leads to Fibrosis, Inflammasome Activation, and Gluconeogenesis Impairment in the Liver of Mice

**DOI:** 10.3390/biology10020082

**Published:** 2021-01-22

**Authors:** Isabela A. Finamor, Caroline A. Bressan, Isabel Torres-Cuevas, Sergio Rius-Pérez, Marcelo da Veiga, Maria I. Rocha, Maria A. Pavanato, Salvador Pérez

**Affiliations:** 1 Department of Physiology and Pharmacology, Federal University of Santa Maria, Santa Maria 97105900, Rio Grande do Sul, Brazil; isabela.finamor@gmail.com (I.A.F.); carolineazzolin@gmail.com (C.A.B.); 2 Neonatal Research Group, Health Research Institute La Fe, 46026 Valencia, Spain; maria.i.torres@uv.es; 3 Department of Physiology, Faculty of Pharmacy, University of Valencia, 46100 Burjassot, Valencia, Spain; sergio.rius@uv.es; 4 Department of Morphology, Federal University of Santa Maria, Santa Maria 97105900, Rio Grande do Sul, Brazil; marceloveiga@gmail.com (M.d.V.); bebelugalde@gmail.com (M.I.R.)

**Keywords:** aspartame, liver fibrosis, lipid peroxidation, Nrf2, inflammasome, PGC-1α, lipid, hypoglycemia, gluconeogenesis

## Abstract

**Simple Summary:**

Aspartame is an artificial sweetener used in foods and beverages worldwide to prevent increasing obesity and diabetes mellitus, acting as a tool to help the control of caloric intake. However, its chronic intake is controversial since it has been linked to some adverse effects including oxidative stress, inflammation, and liver damage through mechanisms that are not fully elucidated yet. Thus, this work aimed to investigate the effects of long-term administration of aspartame on the oxidative and inflammatory mechanisms associated with liver fibrosis progression in mice. Aspartame generated liver injury and fibrosis. It also decreased the activity of antioxidant enzymes and increased the levels of lipid peroxidation, thus, probably, triggering inflammation and cell death through the induction of protein 53 (p53). Finally, via p53 activation, aspartame inhibited a transcriptional coactivator, the peroxisome proliferator-activated receptor gamma coactivator 1 alpha, a master regulator of glucose and lipid metabolism, probably leading to changes in lipid profile in serum, total lipid accumulation, as well as an impairment in the gluconeogenesis in mouse liver, thus causing hypoglycemia. Therefore, this study provides new insights to understand the mechanisms related to aspartame-linked adverse effects, showing that its intake should be cautioned.

**Abstract:**

Background: Aspartame is an artificial sweetener used in foods and beverages worldwide. However, it is linked to oxidative stress, inflammation, and liver damage through mechanisms that are not fully elucidated yet. This work aimed to investigate the effects of long-term administration of aspartame on the oxidative and inflammatory mechanisms associated with liver fibrosis progression in mice. Methods: Mice were divided into two groups with six animals each: control and aspartame. Aspartame (80 mg/kg, via oral) or vehicle was administrated for 12 weeks. Results: Aspartame caused liver damage and elevated serum transaminase levels. Aspartame also generated liver fibrosis, as evidenced by histology analysis, and pro-fibrotic markers’ upregulation, including transforming growth factor β 1, collagen type I alpha 1, and alpha-smooth muscle actin. Furthermore, aspartame reduced nuclear factor erythroid 2-related factor 2 (Nrf2) activation and enzymatic antioxidant activity and increased lipid peroxidation, which triggered NOD-like receptor containing protein 3 (NLRP3) inflammasome activation and p53 induction. Furthermore, aspartame reduced peroxisome proliferator-activated receptor gamma coactivator 1 alpha (PGC-1α) levels, possibly through p53 activation. This PGC-1α deficiency could be responsible for the changes in lipid profile in serum, total lipid accumulation, and gluconeogenesis impairment in liver, evidenced by the gluconeogenic enzymes’ downregulation, thus causing hypoglycemia. Conclusions: This work provides new insights to understand the mechanisms related to the adverse effects of aspartame on liver tissue.

## 1. Introduction

Aspartame is a dipeptide derivative (L-aspartyl L-phenylalanine methyl ester) used in foods and beverages worldwide to prevent increasing obesity and diabetes mellitus, acting as a tool to help the control of caloric intake [1]. After its oral ingestion, aspartame is absorbed from the intestinal lumen and hydrolyzed to phenylalanine (50%), aspartic acid (40%), and methanol (10%) [2]. Although the Food and Drug Administration (FDA) has approved its intake, it has been considered controversial. Indeed, several studies have shown that aspartame consumption is linked to adverse effects in the liver, including inflammation [3,4,5,6,7] and chronic injury [3,8,9]. However, the mechanisms related to these changes have not been fully elucidated until now.

Chronic liver diseases, including liver fibrosis, represent a concern for public health worldwide, with 850 million people affected and a mortality rate of two million deaths per year [10]. Liver fibrosis is a dynamic integrated cellular response to chronic liver injury. It is characterized by loss of parenchymal structure, progressive accumulation of fibrillar extracellular matrix associated with regeneration, and organ dysfunction. Unfortunately, if untreated, liver fibrosis may progress to cirrhosis, hepatocarcinoma, and even death [11].

Oxidative stress can modulate the inflammatory signaling pathways responsible for the progression of liver fibrosis [12]. The decrease in antioxidant defenses during liver damage has been linked to the infiltration of inflammatory cells, the induction of pro-inflammatory cytokines, and the activation of hepatic stellate cells (HSCs) and Kupffer cells that can contribute to the progression of liver fibrosis [13,14]. On the other hand, recent studies have supported that HSCs and Kupffer cells’ stimulation in liver fibrosis may be mediated in part by inflammasome assembly [15,16]. Furthermore, oxidative stress has also been considered fundamental to the specific activation of NOD-like receptor containing protein 3 (NLRP3) inflammasome, the most fully characterized inflammasome complex [17]. Indeed, NLRP3 inflammasome blockades in mice through a mechanism dependent on reactive oxygen species (ROS) can reduce liver inflammation, fibrosis, and hepatocyte pyroptotic cell death [18]. Recently, previous research from our group reported that long-term intake of aspartame triggers reduced glutathione (GSH) depletion in mouse liver due to a blockade in the trans-sulphuration pathway [6]. Therefore, it seems that the oxidative stress associated with aspartame intake could be favoring inflammasome activation. Based on this background, the current work aimed to examine the effects of long-term administration of aspartame on the oxidative and inflammatory mechanisms associated with liver fibrosis progression in mice.

## 2. Materials and Methods

### 2.1. Animals

Male Swiss mice weighing approximately 30 g and aged three months old were obtained from the Biotério Central of the Universidade Federal de Santa Maria (UFSM, Santa Maria, Brazil). They were kept in temperature- and humidity-controlled animal quarters under a 12-h light–dark cycle. The mice were fed a standard rodent chow (Supra, São Leopoldo, Brazil) and tap water ad libitum. The Comissão de Ética no Uso de Animais of the UFSM approved the research protocol (#001/2015).

### 2.2. Experimental Protocol

Mice (*n* = 12) were divided into two groups with six animals each: control and aspartame. Aspartame (Sigma-Aldrich, St. Louis, MO, USA) at 80 mg/kg (2.5 mL/kg, prepared in 0.9% saline solution) or vehicle was administrated by gavage for 12 weeks. Aspartame was prepared daily before administration. After 12 weeks, the animals were fasted overnight and anesthetized with 3% isofluorane, their blood was collected, and they were euthanized by exsanguination for liver removal. A part of the liver was processed immediately and the rest was frozen at −80 °C for future analysis.

### 2.3. Assays

#### 2.3.1. Transaminases, Glucose, and Lipid Profile

Blood was centrifuged at 1000× *g* for 10 min at room temperature. Serum was used for measuring alanine aminotransferase (ALT), aspartate aminotransferase (AST), and glucose, triglycerides, total cholesterol, and high-density lipoprotein (HDL) by using commercial kits (Labtest, Lagoa Santa, Brazil). Low-density lipoproteins (LDL) were assessed according to Friedewald et al. [19] using the following formula: LDL = total cholesterol−(HDL + triglycerides/5). On the other hand, frozen liver samples were homogenized in phosphate-buffered saline (PBS) at 100 mg/mL for total lipid measurement using the TOTAL LIPIDS kit (Spinreact, Girona, Spain). Results were expressed as mg/g protein.

#### 2.3.2. Histology

A piece of liver was rapidly removed, fixed in 10% formaldehyde (Sigma-Aldrich, St. Louis, MO, USA) for 24 h, and embedded in paraffin (Sigma-Aldrich, St. Louis, MO, USA). Sections were prepared at 6 µm using a microtome and then stained with hematoxylin and eosin for studying general histology (Sigma-Aldrich, St. Louis, MO, USA). A quantitative analysis was performed for the inflammatory infiltrate; for this purpose, images were divided in ninety-six quadrants of 1000 μm^2^ and expressed as number of leukocytes per mm^2^. ImageJ was used with the Grid plug-in to analyze these measurements. Additionally, Masson–Goldner’s trichrome staining (Sigma-Aldrich, St. Louis, MO, USA) was used to better visualize collagenous connective tissue fibers, and Sirius Red staining (Sigma-Aldrich, St. Louis, MO, USA) was utilized to detect the different types of collagen fibers.

#### 2.3.3. RT-qPCR

The other portion of the liver was excised and immediately immersed in RNA-later solution (Ambion, Thermo Fisher Scientific, Waltham, MA, USA) to stabilize the RNA. Trizol (Sigma-Aldrich, St. Louis, MO, USA) was used to isolate total RNA. The Revertaid H Minus First Strand cDNA Synthesis Kit (Thermo Fisher Scientific, Waltham, MA, USA) was utilized to construct complementary DNA (cDNA) for amplification in the PCR assay. RT-qPCR was performed in an iQ^TM^5 real-time PCR detection system (BioRad, Hercules, CA, USA). The threshold cycle (CT) was determined, and the relative gene expression was expressed as follows: fold change = 2^−Δ (ΔCT)^, where ΔCT = CTtarget − CThousekeeping, and Δ(ΔCT) = ΔCTtreated − ΔCTcontrol. The specific primers are shown in Table 1, and the TaqMan^TM^ probes (Thermo Fisher Scientific, Waltham, MA, USA) are exhibited in Table 2.

#### 2.3.4. Western Blotting

Frozen liver samples were homogenized in extraction buffer (100 mg/mL) on ice. The extraction buffer contained 20 mM Tris-HCl (pH 7.5), 1 mM ethylenediaminetetraacetic acid (EDTA), 150 mM NaCl, 0.1% SDS, 1% Igepal, 1 mM dithiothreitol, 30 mM sodium pyrophosphate, 50 mM sodium fluoride, 1 mM sodium orthovanadate, and a protease inhibitor cocktail at 0.4%. All reagents were purchased from Sigma-Aldrich, St. Louis, MO, USA. Homogenates were centrifuged at 15,000× *g* for 15 min at 4 °C. The supernatants were used in the assay. Protein extracts (30 µg) were separated in Criterion^®^ Precast Gel 4–15% (BioRad, Hercules, CA, USA) by electrophoresis and transferred to Trans-Blot^®^ Turbo Nitrocellulose membranes (BioRad, Hercules, CA, USA). The non-specific protein-binding sites were blocked with 5% bovine serum albumin (Sigma-Aldrich, St. Louis, MO, USA). The membranes were revealed through chemiluminescence by utilizing Pierce^®^ ECL Western Blotting Substrate (Thermo Fisher Scientific, Waltham, MA, USA) through a ChemiDoc^®^ XRS + System (Biorad, Hercules, CA, USA). The membranes were managed for immunodetection using the following antibodies: anti-alpha smooth muscle actin (α-SMA) (Cell Signaling Technology, Danvers, MA, USA), anti-nuclear factor κB (NF-κB) phospho-p65 (Ser536) (Cell Signaling Technology, Danvers, MA, USA), anti-NF-κB p65 (Cell Signaling Technology, Danvers, MA, USA), anti-NLRP3 (Cell Signaling Technology, Danvers, MA, USA), anti-cleaved caspase 1 (Cell Signaling Technology, Danvers, MA, USA), anti-p53 (Cell Signaling Technology, Danvers, MA, USA), glucose 6-phosphatase (G6Pase) (Abcam, Cambridge, UK), anti-proliferator-activated receptor α coactivator (PGC)-1α (Cell Signaling Technology, Danvers, MA, USA), β-tubulin (Abcam, Cambridge, UK), and anti-Glyceraldehyde-3-phosphate dehydrogenase (GAPDH) (Cell Signaling Technology, Danvers, MA, USA).

#### 2.3.5. Malondialdehyde

Lipid peroxidation was assessed by malondialdehyde measurement (MDA). The supernatant (50 µL) of tissue homogenate (100 mg/mL) was mixed with 500 µL TBA (0.2%)–AcOH 2M (pH 3.5). Then, samples were incubated for 60 min at 95 °C. After cooling, the precipitate was removed by centrifugation at 1000× *g* for 10 min. The reaction product (TBA2–MDA complex) was measured through spectrophotometry. Results were expressed as nmol/mg of protein.

#### 2.3.6. Antioxidant Enzymes

Frozen liver samples were homogenized as described by Da Rosa et al. [20]. Superoxide dismutase (SOD) was assayed as defined by Misra and Fridovich [21] through spectrophotometry. Results were expressed as units of SOD (USOD)/mg protein. One SOD unit was defined as the amount of the enzyme required for 50% inhibition of the adrenochrome formation. Catalase (CAT) was assessed as reported by Aebi [22] through spectrophotometry. Results were expressed as pmol/mg protein. Glutathione peroxidase (GPx) was assayed as described by Flohé and Günzler [23] through spectrophotometry. Results were expressed as µmol/min/mg protein.

#### 2.3.7. Myeloperoxidase

Frozen liver samples were homogenized as defined by Lopez-Front et al. [24]. Myeloperoxidase (MPO) was assessed as reported by Pulli et al. [25]. Results were expressed as mU/mg protein. One MPO unit was defined as the amount of oxidized 3,3′,5,5′-tetramethylbenzidine in 1 min.

#### 2.3.8. Protein

Protein was detected using the Pierce^TM^ BCA Protein Assay Kit (Thermo Fisher Scientific, Waltham, MA, USA).

### 2.4. Statistical Analysis

Statistical analysis was performed using Prism 7.0 (GraphPad Software, San Diego, CA, USA). The data were compared through the Student’s *t*-Test. Results were reported as mean ± standard deviation and the differences were significant at *p* < 0.05.

## 3. Results

### 3.1. Aspartame Leads to Liver Injury

**Figure 1:**
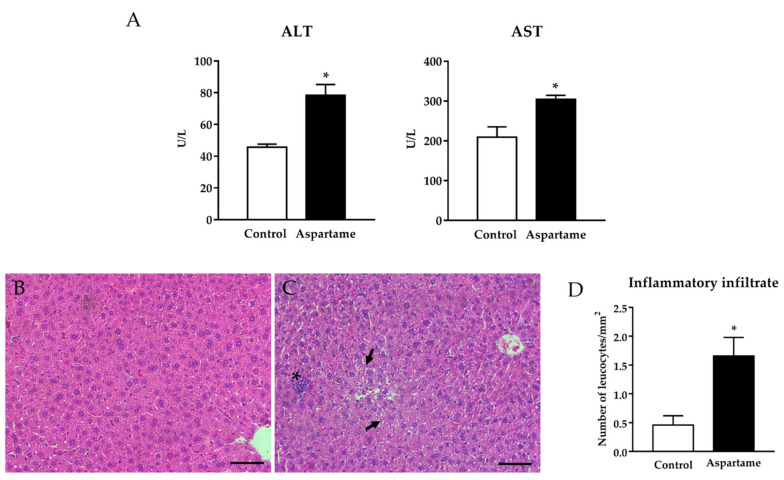
Alanine aminotransferase (ALT) and aspartate aminotransferase (AST) levels (**A**) were measured by spectrophotometry in the sera of control and aspartame-treated mice (*n* = 6, each group). Representative images of hematoxylin–eosin histological staining in the liver of control (**B**) and aspartame-treated mice (**C**). The asterisk indicates inflammatory infiltration while the arrow reveals a reduction in the nuclear volume and hepatocyte degeneration (*n* = 4, each group). Scale bar = 50 µm. Quantitative analysis of inflammatory infiltrate found in liver sections of mice belonging to the different experimental groups (*n* = 4, each group) (**D**). The statistical difference is indicated as follows: * *p* < 0.05 versus control.

ALT and AST levels were increased in the serum of aspartame-treated mice when compared to the control ones (Figure 1A). Additionally, aspartame led to hepatocellular injury, leukocyte infiltration, reduction in the nuclear area, hepatocytes’ degeneration (Figure 1C), and inflammatory infiltration (Figure 1C, quantification in Figure 1D) in different areas of the mice livers. Most features were almost absent in the liver of controls animals (Figure 1B, quantification of inflammatory infiltration in Figure 1D).

### 3.2. Aspartame Causes Liver Fibrosis

**Figure 2:**
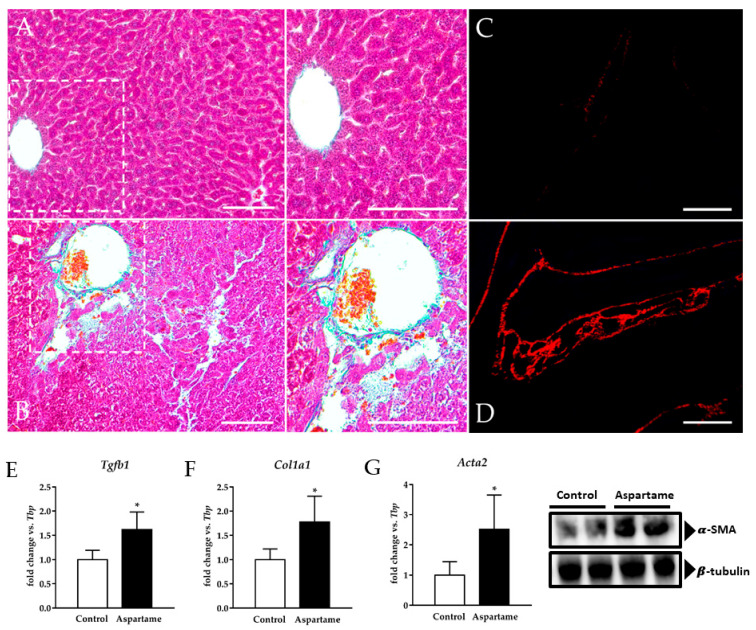
Representative images of Masson–Goldner’s trichrome and Sirius Red staining in the livers of control (**A** and **C**, respectively) and aspartame-treated mice (**B** and **D**, respectively) (*n* = 4, each group). Scale bar = 100 µm. mRNA relative expression of transforming growth factor β 1 (*Tgfb1*) (**E**), collagen type I alpha 1 (*Col1a1*) (**F**), and alpha smooth muscle actin (*Acta2*) (**G**) versus *Tbp* measured by RT-qPCR in the livers of control and aspartame-treated mice (*n* = 6, each group). Representative image of Western blotting of alpha smooth muscle actin (α-SMA) in the livers of control and aspartame-treated mice (*n* = 4, each group). β-tubulin was used as a loading control (**D**). The statistical difference is indicated as follows: * *p* < 0.05 versus control.

Based on the fact that aspartame intake generated injury in the mice liver, we investigated fibrosis through a histological analysis and also by measuring the gene expression of pro-fibrotic markers by RT-qPCR. Aspartame caused remarkable fibrosis as can be evidenced through the much higher deposition of collagen fibers in the mice liver (Figure 2B,D) than in the liver of control animals, where it was found to be almost absent (Figure 2A,C). Furthermore, transforming growth factor β 1 (*Tgfb1*) (Figure 2E), collagen type I alpha 1 (*Col1a1*) (Figure 2F), and alpha smooth muscle actin (*Acta2*) (Figure 2G) mRNA as well as α-SMA protein levels (Figure 2G, densitometry in Figure S1A) were upregulated in the mice livers upon aspartame treatment compared to the control animals.

### 3.3. Aspartame Triggers Lipid Peroxidation and Decreases the Activity of Antioxidant Enzymes

**Figure 3:**
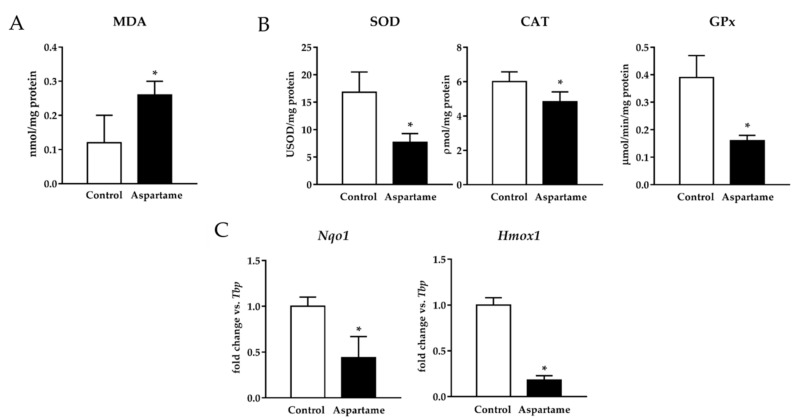
Malondialdehyde (MDA) levels were measured in the livers of control and aspartame-treated mice (*n* = 6, each group) (**A**). The activity of antioxidant enzymes superoxide dismutase (SOD), catalase (CAT), and glutathione peroxidase (GPx) measured by spectrophotometry in the livers of control and aspartame-treated mice (*n* = 6, each group) (**B**). mRNA relative expression of NADPH quinone oxidoreductase 1 (*Nqo1*) and heme oxygenase 1 (*Hmox1*) versus *Tbp* measured by RT-qPCR in the livers of control and aspartame-treated mice (*n* = 6, each group) (**C**). The statistical difference is indicated as follows: * *p* < 0.05 versus control.

Liver fibrosis has been related to oxidative stress [26,27]. For this reason, we checked the MDA levels and the activity of antioxidant enzymes. Aspartame triggered an elevation in the MDA levels (Figure 3A). It was associated with a decrease in the activity of SOD, CAT, and GPx in treated mice livers when compared to the control ones (Figure 3B).

To assess whether nuclear factor erythroid 2-related factor 2 (Nrf2) was involved in the poor antioxidant response induced by aspartame, its targets were measured by RT-qPCR. mRNA NADPH quinone oxidoreductase 1 (*Nqo1*) and heme oxygenase 1 (*Hmox1*) levels were downregulated in the mice liver upon aspartame treatment when compared to the control animals (Figure 3C).

### 3.4. Aspartame Promotes Phosphorylation of p65 Subunit of NF-κB and NLRP3 Inflammasome Activation

**Figure 4:**
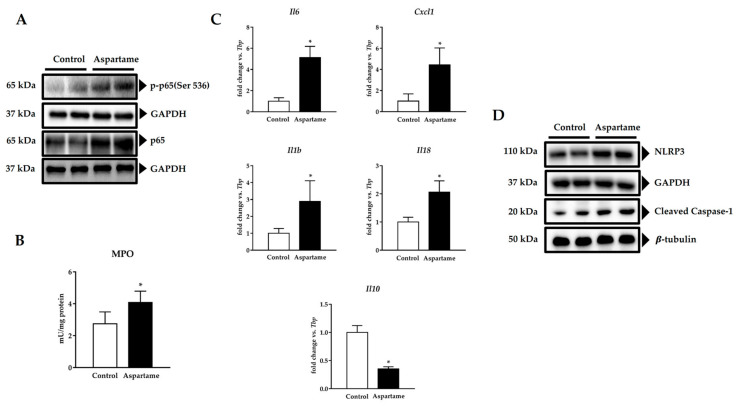
Representative image of Western blotting of phospho-p65 (Ser536) and p65 in the livers of control and aspartame-treated mice (*n* = 4, each group). Glyceraldehyde-3-phosphate dehydrogenase (GAPDH) was used as a loading control (**A**). Myeloperoxidase (MPO) activity in the livers of control and aspartame-treated mice (*n* = 6, each group) (**B**). mRNA relative expression of cytokines interleukin (IL) 6 *Il6*, chemokine (C-X-C motif) ligand 1 (*Cxcl1*), *Ilb1*, *Il18*, and *Il10* versus *Tbp* measured by RT-qPCR in the livers of control and aspartame-treated mice (*n* = 6, each group) (**C**). Representative images of Western blotting of NOD-like receptor containing protein 3 (NLRP3) and cleaved caspase 1 in the livers of control and aspartame-treated mice (*n* = 4, each group). GAPDH and β-tubulin were used as the loading controls, respectively (**D**). The statistical difference is indicated as follows: * *p* < 0.05 versus control.

To evaluate whether NF-κB was also implicated in the poor antioxidant response triggered by aspartame, phosphorylation of its p65 subunit was detected by Western blotting. Phospho-p65 (Ser536) and p65 protein levels were augmented in the livers of aspartame-treated mice when compared to the control ones (Figure 4A, densitometry in Figure S1B).

MPO activity was measured in the mice livers as a marker of inflammatory infiltration. Its activity was higher in the mice livers upon aspartame treatment when compared to the control animals (Figure 4B). Additionally, as NF-κB generally controls the expression of pro-inflammatory genes [28], we determined them by RT-qPCR. Aspartame upregulated the mRNA levels of the pro-inflammatory cytokines interleukin (IL) 6 (*Il6*), chemokine (C-X-C motif) ligand 1 (*Cxcl1*) (equivalent to *Il8*), *Il1b*, and *Il18* and downregulated the mRNA levels of the anti-inflammatory cytokine *Il10* in the treated mice livers when compared to the control ones (Figure 4C).

CXCL1 has been related to NLRP3 inflammasome activation [29]. For this reason, we investigated its levels. Aspartame-treated mice exhibited higher NLRP3 and cleaved caspase 1 protein levels in their livers than the control animals (Figure 4D, densitometry in Figure S1C,D, respectively).

### 3.5. Aspartame Induces p53

**Figure 5:**
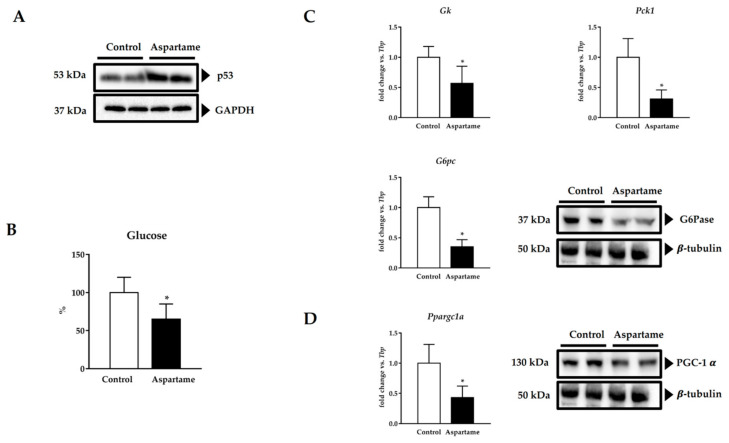
Representative images of Western blotting of p53 in the liver of control and aspartame-treated mice (*n* = 4, each group). GAPDH was used as a loading control (**A**). Fasting glucose levels were measured by spectrophotometry in the serum of control and aspartame-treated mice (*n* = 6, each group) (**B**). mRNA relative expression of gluconeogenic enzymes *Gk*, *Pck1*, *G6pc* versus *Tbp* measured by RT-qPCR (*n* = 6, each group) and representative image of Western blotting of G6Pase in the liver of control and aspartame-treated mice (*n* = 4, each group). β-tubulin was used as a loading control (**C**). mRNA relative expression of *Ppargc1a* versus Tbp measured by RT-qPCR (*n* = 6, each group) and representative image of Western blotting of PGC-1α in the liver of control and aspartame-treated mice (*n* = 4, each group). β-tubulin was used as a loading control (**D**). The statistical difference is indicated as follows: * *p* < 0.05 versus control.

Besides inflammasome activation, apoptosis has also been involved in liver fibrosis via p53 activation [30,31]. Protein levels of p53 were increased in the mice livers upon aspartame treatment when compared to the control ones (Figure 5A, densitometry in Figure S1E).

### 3.6. Aspartame Reduces Fasting Glucose Levels and Impairs Gluconeogenesis via PGC-1α

As shown in Figure 5B, aspartame reduced the levels of fasting glucose in the mice serum compared to the control animals. For this reason, we decided to assess liver gluconeogenesis. Aspartame-treated mice showed lower glycerol kinase (*Gk*), phosphoenolpyruvate kinase (*Pck1*), and both glucose 6-phosphatase mRNA (*G6pc*) and protein (G6Pase) levels in their livers than the control ones (Figure 5C, densitometry in Figure S1F). PGC-1α has been linked to gluconeogenesis regulation [32,33,34]. Moreover, its deficiency has been associated with p53 activation [35]. Therefore, we checked its levels. Aspartame decreased *Ppargc1a* mRNA and PGC-1α protein levels in the mice liver compared to the control animals (Figure 5D, densitometry Figure S1G).

### 3.7. Aspartame Affects the Lipid Profile and Generates the Accumulation of Total Lipids in Liver

PGC-1α has also been related to the regulation of lipid homeostasis [35,36]. So, we determined the lipid profile, observing higher levels of fasting triglycerides, total cholesterol and LDL-cholesterol in serum of fasting aspartame-treated animals than in the control ones (Table 3). On the other hand, aspartame decreased HDL-cholesterol levels in the mice serum when compared to the control ones (Table 3). Moreover, we checked the total lipids in the mice liver, noting higher levels in aspartame-treated animals than in the control mice (Table 3).

## 4. Discussion

Several studies have reported that chronic aspartame intake is associated with liver injury and inflammation in rodents [3,4,5,7]. Accordingly, our group’s recent work showed that aspartame triggered inflammation in the mouse liver, characterized by leukocyte infiltration and hepatocellular injury due to a decrease in the reduced glutathione synthesis through blocking of the trans-sulphuration pathway [6]. Now, for the first time, our data reveal that chronic administration of aspartame to mice induces NLRP3 inflammasome activation and liver fibrosis in a mechanism mediated by oxidative stress. Furthermore, also for the first time, we exhibit that aspartame produces impaired hepatic gluconeogenesis due to PGC-1α downregulation, leading to fasting hypoglycemia.

Liver fibrosis is a sustained wound-healing process that, besides inflammation, involves excessive synthesis and subsequent collagen fiber deposition [37]. Alleva et al. [38] observed that aspartame triggered oxidative stress, release of IL-6, vascular endothelial growth factor A (VEGFA), and their soluble receptors, and mitogen-activated protein kinases’ activation, including ERK and p38, thus creating favorable conditions for inflammatory diseases. Furthermore, it has been shown through histopathologic studies that aspartame can lead to liver fibrosis in rats [3,8,9]. Accordingly, we also revealed that aspartame can generate a much higher deposition of collagen fibers in the liver of mice, probably as a consequence of the upregulation of the pro-fibrotic factors *Tgfb1*, *Col1a1*, and *Acta2* and an increase in the expression of the protein α-SMA. As Del Campo et al. showed [39], severe hepatic fibrosis can progress to liver cancer. Indeed, it was shown that administration of aspartame at high doses (250 and 1000 mg/kg) for eight weeks produced remarkable liver fibrosis and changes in the mRNA levels of crucial oncogenes and tumor suppressor genes, including Harvey rat sarcoma viral oncogene homolog upregulation and P27 downregulation, respectively, indicating its potential risk in progressing to liver cancer [8]. Nevertheless, we did not assess it in this paper, carcinogenesis being an exciting point to be explored in future works.

Oxidative stress has been recognized as a critical factor in the progression of many chronic liver diseases, including liver fibrosis [26,27]. ROS can disturb liver-specific cells’ functionality and, therefore, contribute to the development of liver fibrosis [40]. Several studies have indicated that the liver’s fibrotic disease process is characterized by a decrease in the functioning of the antioxidant defense system associated with Nrf2 deficiency [41]. It was shown that Nrf2 deletion promoted marked oxidative stress, inflammation, and fibrosis in mouse liver [42,43]. On the other hand, it was also reported that Nrf2 activation inhibited the early stage of hepatic fibrosis, reducing Col1a1 and α-SMA levels [44].

Interestingly, it has been shown that aspartame can reduce the activity of antioxidant enzymes such as SOD, CAT, and GPx and increase the levels of lipid peroxidation in rodent liver [45,46]. Furthermore, pronounced lipid peroxidation levels have been detected in biopsies of patients with chronic liver diseases [47]. Similarly, we revealed that aspartame decreased enzymatic antioxidant activity, consequently elevating the mouse liver’s MDA levels. As mentioned before, previous research from our group showed that aspartame caused GSH depletion in mouse liver due to downregulation of the catalytic subunit of glutamate-cysteine ligase levels in mRNA, an Nrf2 target [6]. Now, we observed a downregulation in the mRNA levels of the other two Nrf2 targets, *Nqo1* and *Hmox1*, thus suggesting that aspartame can reduce Nrf2 activity and, therefore, increase lipid peroxidation due to a reduction in the Nrf2-related antioxidant defense in mouse liver.

Liver fibrosis is a complex process involving multiple inflammatory signaling cascades [48]. HSCs are considered the most relevant sources of hepatic myofibroblasts involved in liver fibrogenesis [49], and oxidative stress is critical in HSC stimulation through activation of NF-κB during this process [50]. Besides HSCs, Kupffer cells and infiltrating immune cells have also been linked to liver fibrosis development [51]. In fact, it was shown that leukocyte-derived MPO can induce hepatocyte death through induction of CXCL1 and activation of TGF-β in HSCs [52]. It is noteworthy that aspartame can cause hepatocellular injury in rats by activating Kupffer cells that secrete inflammatory mediators such as IL-6 and/or CXCL1 [7]. Accordingly, we also observed that aspartame upregulated mRNA levels of *Il6*, *Cxcl1*, *Il1b*, and *Il18* in mouse liver. It was linked to an increase in MPO activity and p65 phosphorylation; thus, we suggest that both changes could probably be responsible for cytokine induction. It has recently been described that CXCL1 may sustain a pro-inflammatory state by activating NLRP3 inflammasome in macrophages to intensify the immune response through caspase 1 activation and subsequent IL-1β and IL-18 production and secretion [29]. On the other hand, it is essential to emphasize that aspartame metabolism generates methanol, releasing significant amounts of ROS [53], which can also trigger inflammasome activation [54]. Strikingly, accumulating evidence has shown that NLRP3 inflammasome levels are increased during experimental liver fibrosis, where they are prominently expressed in Kupffer cells and HSCs [18,55]. For the first time, the current research revealed that the aspartame-induced inflammatory response in mouse liver also involved the induction of NLRP3 inflammasome and caspase 1 activation. 

Besides pyroptosis, apoptosis has also been implicated in liver fibrosis development through different p53-related signaling pathways [30,31]. Furthermore, induction of NLRP3 inflammasome has been shown to promote p53 activation and subsequent cell death in a mechanism dependent on DNA oxidation [56]. For this reason, we decided to check the p53 protein levels in the mouse liver, observing that aspartame increased its expression. Thus, it allowed us to postulate that aspartame could have provoked the induction of the mouse liver’s fibrotic factors via p53 activation. Previously, Collison et al. [57] reported that aspartame also promoted the induction of mRNA *p53* levels in mouse liver. However, it resulted in hepatic steatosis, causing less harmful damage than fibrosis. Hence, we believe that the different aspartame doses and routes of administration used by both studies (40 mg/kg in drinking water versus 80 mg/kg via intragastric) were probably responsible for the differing degrees of liver injury since the exposure times were quite similar.

Sahin et al. [58] revealed that p53 represses PGC-1α, impairing mitochondrial biogenesis and function, antioxidative defense, and glucose and lipid metabolism. Based on this fact, we chose to test whether aspartame could change PGC-1α expression in mouse liver, verifying that, indeed, it decreased not only mRNA *Ppargc1a* levels but also protein PGC-1α expression. Besides PGC-1α inhibition, we also detected changes in fasting glucose and lipid profiles in the mice sera. As expected, aspartame promoted an increase in triglyceride, total cholesterol, and LDL cholesterol levels in mouse serum and also a decrease in HDL cholesterol levels as well as an accumulation of lipids in liver tissue [46]. Regarding carbohydrate metabolism, it is interesting to highlight that this is the first report showing that aspartame can reduce fasting glucose levels, since most studies have demonstrated that it can trigger hyperglycemia [3,57,59,60]. Consequently, it prompted us to consider why aspartame occasioned it; therefore, we next decided to investigate the gluconeogenesis pathway in mouse liver since it has been associated with PGC-1α dysfunction [33,34,58].

Gluconeogenesis is one of the main mechanisms by which the liver produces glucose. It involves de novo synthesis of glucose from alanine, lactate, pyruvate, and glycerol, and it is controlled by three critical enzymes, including G6Pase, phosphoenolpyruvate kinase, and fructose-1,6-bisphosphatase [61]. Koo et al. [34] exhibited that PGC-1-deficient mice experienced fasting hypoglycemia linked to the downregulation of mRNA *Pck1* and *G6pc* levels, suggesting that PGC-1α-dependent activation of gluconeogenesis in the liver can be crucial for glucose homeostasis during fasting. For the first time, the current research showed that aspartame inhibited mRNA *Gk*, *Pck1*, and *G6pc* levels and protein G6Pase expression, impairing gluconeogenesis and consequently reducing fasting glucose levels, indicating that perhaps it occurred due to PGC-1α failure.

## 5. Conclusions

In conclusion, aspartame intake decreased enzymatic antioxidant activity, markedly reduced Nrf2 activation, and increased lipid peroxidation levels, thus triggering an inflammatory response that caused NLRP3 inflammasome activation in mouse liver. Aspartame also generated liver fibrosis, evidenced by the increase in mRNA *Tgfb1*, *Col1a1*, and *Acta2* levels and protein α-SMA expression, probably due to the activation of NLRP3 inflammasome and p53. Furthermore, aspartame may have reduced both mRNA *Ppargc1a* levels and protein PGC-1α expression through p53 activation. This PGC-1α deficiency could be responsible for the changes in the fasting serum lipid profile and the accumulation of total lipids as well as the gluconeogenesis impairment in the mouse liver model, evidenced by the downregulation of mRNA *Gk*, *Pck1*, and *G6pc* levels and protein G6Pase expression, thus causing decreased levels of fasting glucose. Finally, this work provides new insights to understand the mechanisms related to aspartame-linked adverse effects.

## Figures and Tables

**Table 1:** The oligonucleotides used for RT-qPCR.

Target Gene (mm)	Direct/Reverse Oligonucleotide
*Acta2* (Gene ID: 11475)	F→ TTCATTGGGATGGAGTCAGC R→ CTTCTGCATCCTGTCAGCAA
*Col1a1* (Gene ID: 12842)	F→ GCCAAGAAGACATCCCTGAA R→ GGTTGGGACAGTCCAGTTCT
*G6pc* (Gene ID: 14377)	F→ CAACGTATGGATTCCGGTGT R→ GAAAGTTTCAGCCACAGCAA
*Gk* (Gene ID: 14933)	F→ GTTGGAAGGTTCCGTGGCTA R→ AACCCTGAAAATGCTGGAAC
*Hmox1* (Gene ID: 15368)	F→ AAGAGGCTAAGACCGCCTTC R→ GCCACATTGGACAGAGTTCA
*Nqo1* (Gene ID: 18104)	F→ TTCTCTGGCCGATTCAGAGT R→ GGCTGCTTGGAGCAAAATA
*Pck1* (Gene ID: 18534)	F→ CCAAGGCAACTTAAGGGCTA R→ TAAACACCCCCATCGCTAGT
*Ppargc1a* (Gene ID: 19017)	F→ TTAAAGTTCATGGGGCAAGC R→ TAGGAATGGCTGAAGGGATG
*Tbp* (Gene ID: 21374)	F→CAGCCTTCCACCTTATGCTC R→ CCGTAAGGCATCATTGGACT
*Tgfb1* (Gene ID: 21803)	F→ TTGCTTCAGCTCCACAGAGA R→ TGGTTGTAGAGGGCAAGGAC

**Table 2:** The TaqMan^TM^ probes used for RT-qPCR.

Target Gene (mm)	TaqMan Probe
*Cxcl1* (Gene ID: 14825)	Mm04207460_m1
*Il1b* (Gene ID: 16176)	Mm00434228_m1
*Il6* (Gene ID: 16193)	Mm00446190_m1
*Il10* (Gene ID: 16153)	Mm01288386_m1
*Il18* (Gene ID: 16173)	Mm00434226_m1
*Tbp* (Gene ID: 21374)	Mm01277042_m1

**Table 3:** Effects of aspartame on fasting lipid profile in serum and total lipids in the mice liver.

	Control	Aspartame
Total Cholesterol (mg/dL)	117.0 ± 16.4	153.0 ± 22.8 *
HDL cholesterol (mg/dL)	37.0 ± 11.8	25.5 ± 12.1 *
LDL cholesterol (mg/dL)	47.6 ± 22.9	84.2 ± 21.5 *
Triglycerides (mg/dL)	120.0 ± 29.0	191.5 ± 27.0 *
Total lipids (mg/g protein)	8.55 ± 3.59	15.02 ± 2.22 *

The number of mice per group was six. The statistical difference is indicated as follows: * *p* < 0.05 versus control.

## Supplementary Materials

The following are available online at https://www.mdpi.com/2079-7737/10/2/82/s1, Figure S1: Quantification of α-SMA (**A**), phospho-p65(Ser536) to p65 (**B**), NLRP3 (**C**), cleaved caspase-1 (**D**), p53 (**E**), G6Pase (**F**) and PGC-1α (**G**) protein levels by Western blotting in the liver of control and aspartame-treated mice (*n* = 4, each group). The statistical difference is indicated as follows: ^*^ *p* <0.05 versus control.
