# Supplementary material for: Long-Term Aspartame Administration Leads to Fibrosis, Inflammasome Activation, and Gluconeogenesis Impairment in the Liver of Mice
Source: Biology (Basel). 2021 Jan 22;10(2):82. doi: 10.3390/biology10020082 (PMC7911935; doi:10.3390/biology10020082)
Supplement: Supplementary file 1 [file biology-10-00082-s001.pdf]

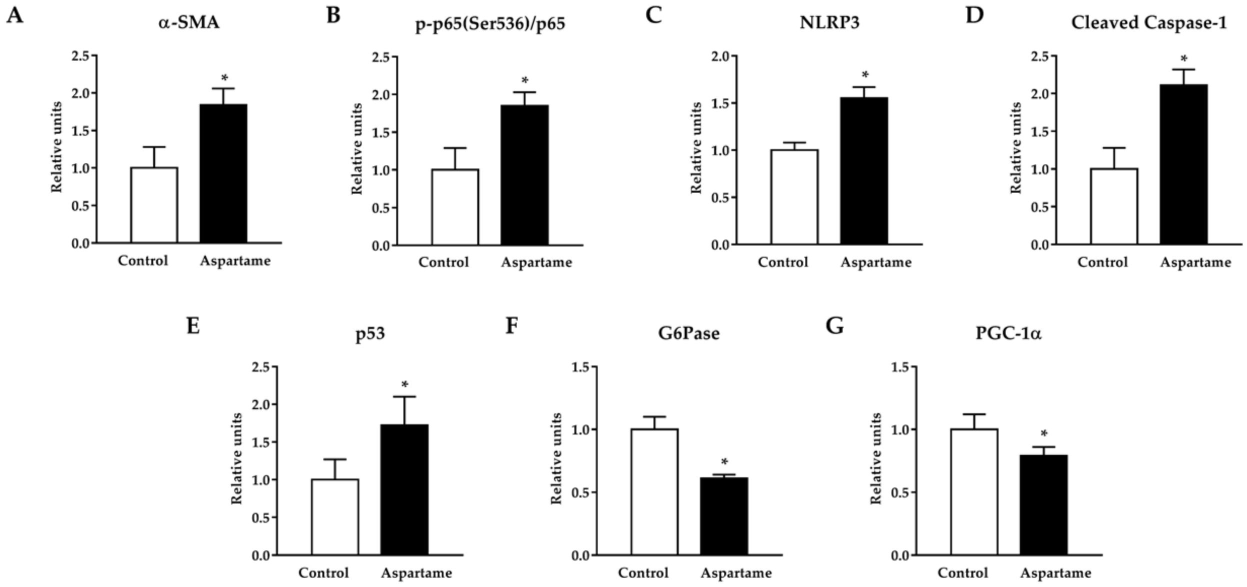

Figure S1. Quantification of α-SMA (A), phospho-p65(Ser536) to p65 (B), NLRP3 (C), cleaved caspase-1 (D), p53 (E), G6Pase (F) and PGC-1α (G) protein levels by Western blotting in the liver of control and aspartame-treated mice (n = 4, each group). The statistical difference is indicated as follows: $^{*}P$ <0.05 versus control.
